# Assessment of energy management and power quality improvement of hydrogen based microgrid system through novel PSO-MWWO technique

**DOI:** 10.1038/s41598-024-78153-4

**Published:** 2025-01-05

**Authors:** Hafiz Ghulam Murtza Qamar, Xiaoqiang Guo, Ehab Seif Ghith, Mehdi Tlija, Abubakar Siddique

**Affiliations:** 1 Key Laboratory of Power Electronics for Energy Conservation and Motor Drive of Hebei Province, Department of Electrical Engineering, Yanshan University, Qinhuangdao, 066004 China; 2 Department of Mechatronics, Faculty of Engineering, Ain shams University, Cairo, 11566 Egypt; 3 Department of Industrial Engineering, College of Engineering, King Saud University, P.O. Box 800, Riyadh, 11421 Saudi Arabia; 4 Department of Electrical & Biomedical Engineering, Khwaja Fareed University of Engineering and Information Technology (KFUEIT), Rahim Yar Khan, 64200 Pakistan

**Keywords:** Hydrogen based microgrid, Energy management system EMS, Power quality, Particle swarm optimization-modified water wave optimization, Engineering, Electrical and electronic engineering

## Abstract

A hybrid microgrid powered by hydrogen is an energy infrastructure that depends on hydrogen as its primary energy carrier within a localized network. This study proposed a novel bi-level optimization approach to enhance power quality and cost efficiency of the system. In the quest to improve energy management systems (EMS) and enhance power quality, a bi-level optimization approach named Particle swarm optimization-Modified water wave optimization (PSO-MWWO) has been proposed. This method integrates adaptive population size and an adaptive wavelength coefficient to boost its overall effectiveness. It is attractive with specialists in energy management systems (EMS), control systems, and hydrogen technologies can significantly augment the efficiency of coordination endeavours. PSO-MWWO can incorporate environmental considerations, such as minimizing emissions or exploiting the use of renewable energy resources (RESs) in hydrogen production and consumption. This paper thoroughly examines its implementation, operation, and unique features, with a particular emphasis on the power quality of a hydrogen based microgrid. The achieved results and numerical analysis affirm the superiority of the proposed technique compared to other traditional methods like mixed integer linear programming (MILP), HOMER, Variable mesh optimization (VMO), and Cataclysmic genetic algorithm in optimizing component sizing, renewable production, hydrogen production, reliability, cost effective, and overall efficacy. This substantiates its practical utility in real-time applications. The efficacy of this method is empirically demonstrated through the implementation in MATLAB software.

**Supplementary Information:**

The online version contains supplementary material available at 10.1038/s41598-024-78153-4.

## Introduction

In the comprehensive context of recent energy systems, microgrids play a key role in endorsing sustainability, consolidation reliability, and progressing energy self-sufficiency. The role becomes principally obvious amid the growing adoption of renewable energy sources and the intensifying demand for robust energy solutions^1^. Microgrids represent decentralized energy systems that operate on a smaller scale once associated to traditional centralized grids^2^. Hybrid microgrids are designed to generate, distribute, and manage power within precise localized areas, such as neighborhoods, commercial complexes,, or even industrial sites^3^. Microgrids encompass numerous decisive features, including their energy sources, ability to operate independently (islanded operation), connectivity to the main grid, energy management strategies, and local control mechanisms^4^. A significant benefit of microgrids is their resilience when confronted with power disruptions^5^. They have the capability to preserve power supply even in the incident of power outages, which is a crucial feature for vigorous services such as hospitals, emergency response centers, and isolated communities^6^. By efficiently managing energy production and consumption at a local level, microgrids contribute to enhanced energy efficacy^7^.

Hybrid grids is an emerging technology and concept that embraces substantial potential for addressing some of the encounters associated with renewable energy integration and grid steadiness^8–10^. Hydrogen is considered an energy carrier and storage key for power grids. It can store surplus energy when supply exceeds demand and consume it when required, helping to balance the grid supply^11^. During periods of low energy production, the stored hydrogen will be converted in a fuel cell, ensuring a dependable power supply to meet load demand^12^. Hydrogen can be produced through numerous approaches, including electrolysis (by means of power to split water into hydrogen and oxygen), steam methane ameliorating, and other progressive technologies. Renewable energy sources like wind and solar can be utilized for green hydrogen production^13^. Hydrogen can be stored in several forms, like gaseous hydrogen, liquid hydrogen, and solid hydrogen storage resources. The storage process choices actually depend on factors like efficiency, safety, and microgrid provisions^14,15^.

Hydrogen’s high energy density makes it ideal for storing substantial amounts of energy in relatively small volumes, which is beneficial for grid-scale storage^16^. Hydrogen storage enables the integration of fragmentary renewable energy sources (such as wind and solar) into the grid by providing a means to store surplus energy and deliver it when these sources are not producing^13^. Previous studies have explored the techno-economic benefits of electrolyzer capacity, hydrogen storage tank size, and the integration of renewable energy in the system^17^. Although hydrogen production is free from greenhouse gas emissions, the combustion of hydrogen, similar to other high-temperature burning processes, releases nitrogen oxides. These pollutants contribute to smog, acid rain, and health issues such as asthma and respiratory infections^18^. Transition to hydrogen could significantly reduce the warming effects associated with fossil fuels, provided that the hydrogen is produced from renewable energy resources and that strict measures are implemented to prevent hydrogen leaks and other secretions^19^. Hydrogen production offers significant potential for decarbonizing various sectors, such as transportation, industry, and power generation^20^. By achieving this, it can play a crucial role in global efforts to reduce greenhouse gas emissions and combat climate change^21^.

**Fig. 1:**
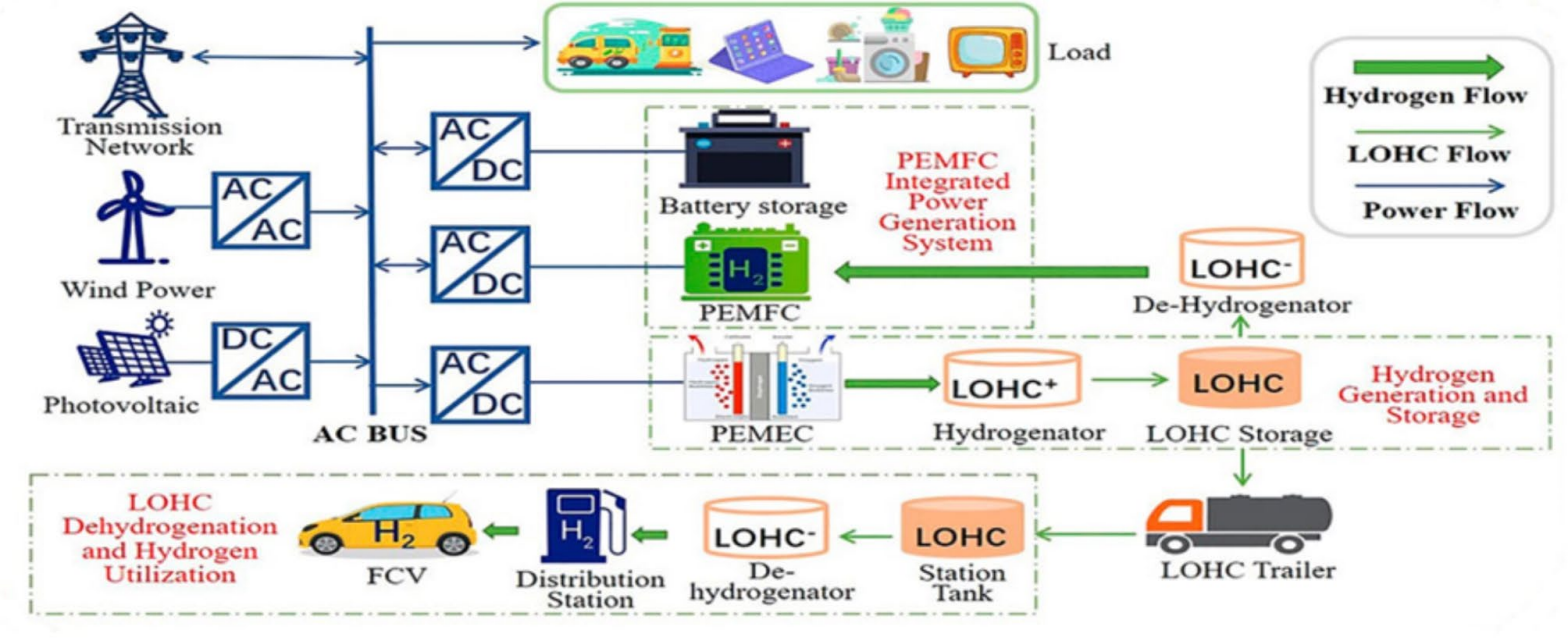
Structure of hydrogen storage based microgrid^23^.

Hydrogen storage can improve grid resilience by providing backup power during grid outages or disasters. Fuel cells that produce power from stored hydrogen can be utilized for this purpose. Hydrogen can be transported over extensive reserves than power, making it a valuable option for connecting remote renewable energy generation sites to the grid or for communicating local grids^22^. The common structure of the microgrid is elaborated in Fig. 1.

However, it’s important to acknowledge that hydrogen storage also faces several challenges, including energy losses during conversion and storage, cost-effectiveness, safety considerations, and the necessity for infrastructure development such as pipelines and storage facilities^16^. Nonetheless, numerous pilot projects and initiatives are underway worldwide to explore and implement hydrogen storage solutions within power grids, marking an exciting advancement in the quest for cleaner and more reliable energy systems. These projects aim to establish the technology’s feasibility and potential benefits. Hydrogen storage in power grids is a chunk of the wider efforts to evolution to cleaner and additional sustainable energy systems. It offers an auspicious resolution to the challenges related to renewable energy unpredictability and grid reliability while contributing to the decarbonization of the energy sector^24^. The systematic challenges associated with hydrogen based microgrids, such as transportation, storage, and distribution issues, along with technical and economic barriers, are addressed^25^.

Modified Water Wave Optimization (MWWO) and Particle Swarm Optimization (PSO) are both nature-inspired optimization techniques used to crack complex optimization hitches in this study. Each of them is based on different ideologies and mechanisms^26,27^. MWWO is exceptional in the behavior of water waves. It imitates the propagation and interaction of waves to compute solutions^28^. PSO is stimulated by the means of an entire bird’s flock or fish school. It simulates the way distinct particles in a swarm collaborate to find the ultimate solution^29^. MWWO characterizes potential solutions as waves on a water surface. These waves propagate and interact with each other, representing the exchange of information among solutions.

Particles regulate their velocities based on their own finest position and most notable position between the complete crowd. MWWO introduces damping factors to control convergence, simulating the dissipation of energy in real water waves. PSO balances exploration (finding new areas of the search space) and exploitation (refining the current best solution). Both PSO and MWWO are part of the extensive field of metaheuristic optimization, and their efficacy depends on features like parameter tuning and the specific characteristics of the problem being solved^30^. This study examines the efficiency of hybrid microgrids, with focus on improving the power quality through cost-effective optimization techniques^31^. Researchers often choose between these algorithms based on the problem at hand and their performance in practice^32–34^. The Energy Management System is established to account for power fluctuations due to uncertainties in renewable energy^35^.

As outlined in the preceding descriptions, the primary advantage of this research lies in the upgraded cost-effectiveness of hydrogen based microgrids through effective energy management and improve the power quality of the system^36^. The utilization of MWWO and PSO to address this model is a noteworthy aspect because these are not explored previously in the field of hydrogen based microgrid applications. Furthermore, the article considers the eventual achievement of voltage convergence in this model to improve the power profile^37^. The proposed research design offers notable contributuion over traditional microgrid design approaches:

- In this paper, bi level nature inspired optimization techniques Particle swarm optimization-Modified water wave optimization (PSO-MWWO) are proposed, it steadily delivers superior results in optimizing component sizing, renewable production, hydrogen production, reliability, cost effective, and overall efficacy when compared to traditional hydrogen based microgrid designs.
- Utilizing hybrid microgrid, the PSO-MWWO approach modernized the assemblage of microgrid anatomy, simplifying the preliminary design development and enhance the system performance due to with its better speed of convergence, greater solution diversity, and improved balance between exploration and exploitation.
- The proposed PSO-MWWO technique converges the power at an appropriate level and improves the power quality ultimately due to some factors like search spaceis increased, optimization characteristics by tying the local and the global exploration aptitude are remarkable.
- To justify the efficiency and strength of suggested PSO-MWWO novel technique over traditional methods, a convergence of power has been implemented which has not been active much in literature. Presenting these comparisons are in table is a novel approach, making them easy to understand and encouraging researcher to pursue further studies in this field of interest.
- The ultimate outcomes showed that the proposed PSO-MWWO technique is robust and outclass the conventional techiques in terms proper energy distribution among the hybrid energy resources, better SoC, lower level of harmoics, maximizing the system efficiency in a cost effective manner and improved voltage response by improving power quality.

Further sections of this article cover: section II the hydrogen based system model, section III described the optimized energy management system EMS, the Simulation and its results are demonstrated in section IV and final section V holds the conclusion.

## Hydrogen based system model

This section delves into modeling the microgrid (MG) system and its components, stressing the importance of accurately representing each subsystem. Employing a meticulous approach, crafted a precise model that includes elements like wind turbine, photovoltaic, hydrogen repository, electrolyzers and fuel cell with variable power. The section conducts a thorough investigation of each component within the system.

This research presents a microgrid system that unified several components, containing a proton exchange membrane fuel cell, an alkaline electrolyzer, oxygen and hydrogen gas storage enclosure, battery storage, and photovoltaic generators^38^. In precise, the fuel cell and electrolyzer utilized in this research align with the specifications of a Nexa 26 V, 46 A, 1.2 kW Proton Exchange Membrane Fuel Cell (PEMFC) established by the Ballard^39^and hydrogen Igen 300/1/25, 43 V, 120 A, 5 kW Alkaline Electrolyzer. To ehnace the global performance and resource usage of hybrid microgrids, a comprehensive power and energy management plan of action is put into practice^40^. This approach is purposefully crafted to optimize the system’s operation^41^. The hydrogen based microgrid is simulated using MATLAB/Simulink software.

At the initial phase in designing a microgrid is to estimate the energy requirements of the load or the capacity it will oblige. It embraces assessing the typical daily and peak energy demand, as well as recognizing the energy sources currently used and the potential for renewable energy. This comprises recognizing the quantity of hydrogen storage, fuel cells, and further components mandatory to encounter the energy demand. This involves choosing the proper fuel cells, power electronics, and other components to optimize the performance of the system. The management of the microgrid system involves monitoring the energy demand and supply, managing the hydrogen production and storage, and maintaining the system components. It requires the development of a control system that can efficiently accomplish the energy flows and optimize the system’s performance.

### Wind turbine model

Wind turbines produce electrical power continuously, delivering energy for local consumption and replenishing a hydrogen storage system. Additional power is either fed back into the grid or used for non-critical purposes. Computing the output power of the wind mills entails transforming the wind speed, as measured at the anemometer height, to measure the wind turbine hub height by means of the power law Eq. (1)^42^.

1 $${\text{V}}_{\text{b}} = {\text{V}}_{\text{a}} ( {\frac{\text{h}} {{\text{h}}_{\rm ref}} ) }^{{\upgamma}}$$

In this scenario, $\:{V}_{a}$ and $\:{V}_{b}$ represents wind speeds at the wind turbine hub height and the reference height $\:{h}_{ref}$, calculated in meters per second. Moreover, γ denotes the exponential factor within the power law and is usually known as the “roughness factor.” This factor is permitting to the impact of various variables, such as the local terrain, temperature, and wind velocity, which can vary with the time of day or time of year. Therefore, it can oscillate significantly between open, flat landscapes and compact forested areas. As a result, the wind turbine’s power output, considered as $\:{P}_{Wout}$, can be determined using a series of Eq. (2).

2 $$\:{\text{P}}_{\text{W}\text{o}\text{u}\text{t}}\:=\:\left\{\begin{array}{c}\begin{array}{c}\left(\text{x}{\text{V}}^{3}-\text{y}\right)\:{\text{P}}_{\text{R}\text{W}},\:\:\:{\text{V}}_{\text{C}-\text{i}\text{n}}\le\:V\le\:\:{\text{V}}_{\text{R}\text{a}}\:\:\:\:\:\:\:\\\:{\text{P}}_{\text{R}\text{W}\:,\:}\:\:\:\:\:\:\:\:\:\:\:\:{\text{V}}_{\text{R}\text{a}}\:\le\:V\le\:\:{\text{V}}_{\text{C}-\text{O}\text{f}\text{f}}\:\:\:\:\:\:\:\:\:\:\:\:\:\:\:\:\:\:\:\:\end{array}\\\:0,\:\:\:\:\:\:\:\:\:\:\:\:\:\:\:\:\:\:\:\:\:\:\:\:\:\:\:\:\:\:\:Otherwise\end{array}\:\right.$$

The output of WT is relying on two key factors: the rated power $\:{P}_{Wout}$ of the turbine and wind speed V. Moreover, vital wind velocity standards are used to control the turbine’s action. The Cut-in velocity presented by $\:{V}_{C-in}$, the rated speed of the turbine designated by $\:{V}_{Ra}$and lastly, the cut-off speed can be determined by $\:{V}_{C-Off}$. So, remaining constant values $\:x$ and $\:y$ can be calculated by specific expressions (3) and (4):

3 $$\:\text{x}\:=\:\frac{1}{{{\text{V}}_{\text{R}\text{a}}}^{3}-\:{{\text{V}}_{\text{C}-\text{i}\text{n}}}^{3}}$$

4 $$\:\text{y}\:=\:\frac{{{\text{V}}_{\text{C}-\text{i}\text{n}}}^{3}}{{{\text{V}}_{\text{R}\text{a}}}^{3}-\:{{\text{V}}_{\text{C}-\text{i}\text{n}}}^{3}}$$

Usually, the $\:{V}_{C-in}$ can be found within the bracket of 2.5–3.5 m per second and $\:{V}_{C-Off}$ has the range of 20–25 m per second.

### Photovoltaic model

During this phase of the article, solar panels produce power in the presence of sunshine^43^. The produced power is employed to meet local energy needs and replenish the hydrogen storage. The surplus electrical power can be directed back to the grid or utilized for non-essential devices. Information necessary for solar power management is sourced from solar irradiation (I) and ambient temperature ($\:{T}_{a}$), which are acquired from the National Solar Radiation Database (NSRDB). When PV power production starts, the cell temperature ($\:{T}_{Cell}$) is computed using a specific (5).

5 $$T_{\rm cell} (t)= T_{a} (t)+I(t)\left(\frac{\text{N}\text{O}\text{C}\text{T}-20}{0.8}\right)$$

Where NOCT represents the nominal operating cell temperature of the solar panels, the output power of a PV cell is computed using (6).

6 $$P_{PV}(t)=(R_{\rm fac}) (\eta_{PV})(A_{PV})(I(t)) \left( 1-\frac{{K}_{P}}{100 \left({T}_{C} (t)-25\right)} \right)$$

$\:{R}_{fac}$ is the reduction factor accounting for dust accretion, on the other hand $\:{\eta}_{PV}$ denotes the solar panels conversion efficiency, $\:{A}_{PV}\:$signifies the surface area of the panels, and $\:{K}_{P}\:$represents the temperature coefficient. As a result, (7) quantifies the total power generated by an entire PV panel.

7 $$P_{\rm total} (t) = (N_{PV}) ({P}_{PV}(t))$$

$\:{P}_{total\:\:}$is the total power and the temperature rise may compromise its efficacy. The minor modification in temperature of solar panels can lead to a decrease in power concerning both voltage and current.

### Hydrogen repository model

The object of a hydrogen storage system is similar to common energy storage concepts. It encloses the production of hydrogen through electrolysis by means of excess power, its storage within the tank, and its assembly to a fuel cell for hydrogen release. The repository capacity of the hydrogen tank has a direct influence on the quantity of hydrogen that can be stored and discharged. This relationship is articulated as follows in (8).

8 $$C_{H_{2}} (t+1)= C_{H_{2}} (t) + k P_{{\rm out} - {H_{2}} } (t) \frac{{\text{P}}_{\text{i}\text{n}-\text{F}\text{C}}\left(\text{t}\right)}{\text{k}}$$

where,$\:{\:\text{C}}_{{\text{H}}_{2}}\left(\text{t}+1\right)$ is the refurbished capacity of the tank, while $\:{\text{C}}_{{\text{H}}_{2}}$(t) is the running capacity of the container and k is the storage efficiency of tank. $\:{\text{P}}_{\text{i}\text{n}-\text{F}\text{C}}\left(\text{t}\right)$ is the power of the hydrogen container at time t.

### Electrolyzer Model

Hydrogen generation is made by employing an electrolyzer, which acts as the primary device for transforming power into hydrogen. The outcomes of the electrolyzers functioning can be succinctly represented by (9)

9 $$\:{\text{P}}_{\text{o}\text{u}\text{t}-{\text{H}}_{2}}\left(\text{t}\right)\:=\:{\text{k}}_{1}{\text{P}}_{\text{i}\text{n}-\text{E}}\left(\text{t}\right)$$

Where, $\:{\text{k}}_{1}$ represents the electro-hydrogen conversion ratio. $\:{\text{P}}_{\text{i}\text{n}-\text{E}}\left(\text{t}\right)$ and $\:{\text{P}}_{\text{o}\text{u}\text{t}-{\text{H}}_{2}}$(t) are the input/ output power of the electrolyzer at the prescribed time t.

### FC Model

When the power supply is inadequate, a fuel cell steps in to produce power by transforming chemical energy into electrical energy. The amount of power generated by fuel cells can be designated as a function of power released from hydrogen and the efficiency with which the hydrogen’s electrical energy is converted. So, the output power can be measured in (10).

10 $$\:{\text{P}}_{\text{o}\text{u}\text{t}-\text{F}\text{C}}\:\left(\text{t}\right)\:=\:{\text{k}}_{1}{\text{P}}_{\text{i}\text{n}-\text{F}\text{C}}\left(\text{t}\right)$$

In the present disclosure the oxygen and hydrogen chunk are to be considered is lossless. The total volume of $\:{\text{O}}_{2}$ and $\:{\text{H}}_{2}$ can be calculated by (11) and (12)

11 $${\text{V}}_{\rm O,t}={\text{V}}_{\rm {O,t}-1}+{\text{n}}_{\text{O},\text{t}}{\text{t}}_{\text{o}\text{p}\text{t}}$$

12 $${\text{V}}_{\rm H,t}={\text{V}}_{\rm {H,t}-1}+{\text{n}}_{\text{H},\text{t}}{\text{t}}_{\text{o}\text{p}\text{t}}$$

The volume of container level variance hinges on the primary level like $\:{\text{V}}_{\text{O},\text{t}}$ and $\:{\text{V}}_{\text{H},\text{t}}$. Flow rates are labelled in $\:{n}_{O,t}{t}_{opt}$ and $\:{n}_{H,t}{t}_{opt}$ respectively. Matlab polyfitZero function can give these terminologies. So, flow rates of oxygen and hydrogen can be measured by (13) and (14)

13 $${\text{n}}_{\text{O,t}} = {\text{n}}_{\text{O El,t}}+ {\text{n}}_{\text{O}\:\text{F}\text{C},\text{t}}$$

14 $${\text{n}}_{\text{H,t}} = {\text{n}}_{\text{H El,t}}+ {\text{n}}_{\text{H}\:\text{F}\text{C},\text{t}}$$

The flow rates of oxygen based electrolyzer and FC are expressed as $\:{\text{n}}_{\text{O}\:\text{E}\text{l},\text{n}}$ and $\:{\text{n}}_{\text{O}\:\text{F}\text{C},\text{n}}$, though the flow rates of the hydrogen based electrolyzer and FC are $\:{\text{n}}_{\text{O}\:\text{E}\text{l},\text{t}}$ and $\:{\text{n}}_{\text{H}\:\text{F}\text{C},\text{t}}$ respectively. Now the exchange current and voltage can be measured by (15–17).

15 $${\text{i}}_{\rm out}=\frac{2\text{F}\text{k}\:({\text{P}}_{{\text{H}}_{2}}+\:{\text{P}}_{{\text{O}}_{2}})}{\text{R}\text{h}} {\text{exp}}\left(\frac{-\Delta \text{G}}{{\text{RT}}}\right)$$

16 $${\text{V}}_{\rm out}={\text{E}}_{\text{O}\text{C}} - {\text{V}}_{\rm Res} - {\text{V}}_{\rm D}$$

17 $${\text{V}}_{\text{F}\text{C}} = {\text{N}} \times {\text{V}}_{\rm out}$$

Where $\:{\text{V}}_{\text{o}\text{u}\text{t}}$ and $\:{\text{i}}_{\text{o}\text{u}\text{t}}$ are output of voltage and current, ‘Rh’ is the plank’s constant and k is Boltzmann constant while N is the number of cells and output voltage taking all losses including $\:{\text{V}}_{\text{R}\text{e}\text{s}}$ resistive and $\:{\text{V}}_{\text{D}}$ deffusive.

## Optimized energy management system

In the strategic development and modeling of hydrogen based microgrids, optimization models are often crafted with a focus on critical metrics that include investment costs, power reliability, and emissions of pollutants. In the hybrid microgrid design presented in this research, the optimization model revolves around pivotal decision variables, capacities of the electrolyzer, hydrogen storage tank, and FC within the hydrogen storage system. This model offers an inclusive assessment, considering aspects like initial cost, present cost, and the efficiency of power shortages linked to the hydrogen storage unit. Eventually, it fabricates an optimized model for the hydrogen storage system, thereby enhancing the precision and efficiency of microgrid planning and modeling.

When deciding on the power conversion structure and the scale of power sources for a microgrid serving crucial loads, there are two distinct performance metrics to contemplate like enhancing the power efficiency of the microgrid by improving voltage profile and the final one is cost-effectiveness. The incorporation of Particle Swarm Optimization (PSO) and Modified Water Wave Optimization (MWWO) within hydrogen based microgrids has the potential to elevate the control of energy resources, boost system efficiency, and promote the utilization of hydrogen as an energy carrier. So, how these optimization algorithms can be applied in the context of hydrogen based microgrids. The objective function of the energy management system, given in (18), is based on two main factors: enhancing power system quality and being cost-effective.

18 $$Obj_{fcn} =[{P}_{\eta}, Cost\_effective]$$

Particle Swarm Optimization (PSO) can efficiently optimize the allocation of hydrogen-based power generation to meet microgrid demand, thereby enhancing power quality and efficiency $\:{P}_{\eta}$ and that can be done when to import/export electricity based on hydrogen availability and improves the voltage profile.

**Fig. 2:**
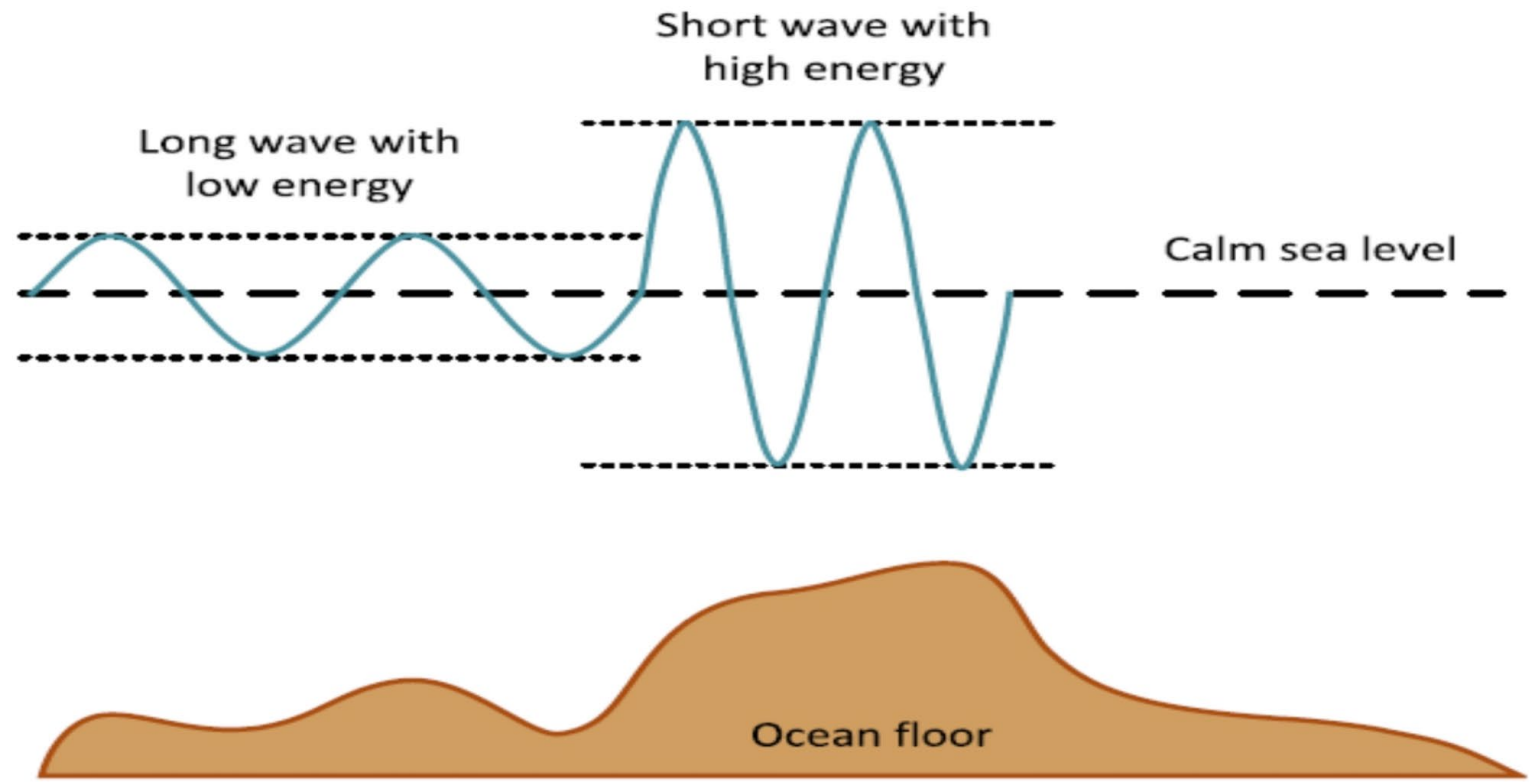
Wave shapes with respect to wave energy^44^.

Modified Water Wave Optimization (MWWO) can be employed to optimize the production of hydrogen considering real-time factors such as weather conditions, renewable energy generation, and demand fluctuations. Modified Water Wave Optimization (MWWO) optimizes energy management within the microgrid by taking into account factors such as hydrogen availability, load patterns, and environmental goals, ensuring optimal operation under changing conditions. A graphical depiction of numerous wave shapes in profound and thin water for the MWWO model is offered in Fig. 2.

Three categories of operation on the waves are painstaking which are termed as propagation, refraction and breaking correspondingly in (19–20)^45^.

19 $${x}^{{\prime}}(t) = x(t) + {\text{rand}} (-1,1) \cdot \lambda L (T)$$

20 $$\:\lambda\:=\:{\lambda\:}_{0}\:{r}^{-\:\frac{(f\left(x\right)-\:{f}_{min}+\epsilon\:)}{({f}_{min}+\:{f}_{max}+\:\epsilon\:)}}$$

**Fig. 3:**
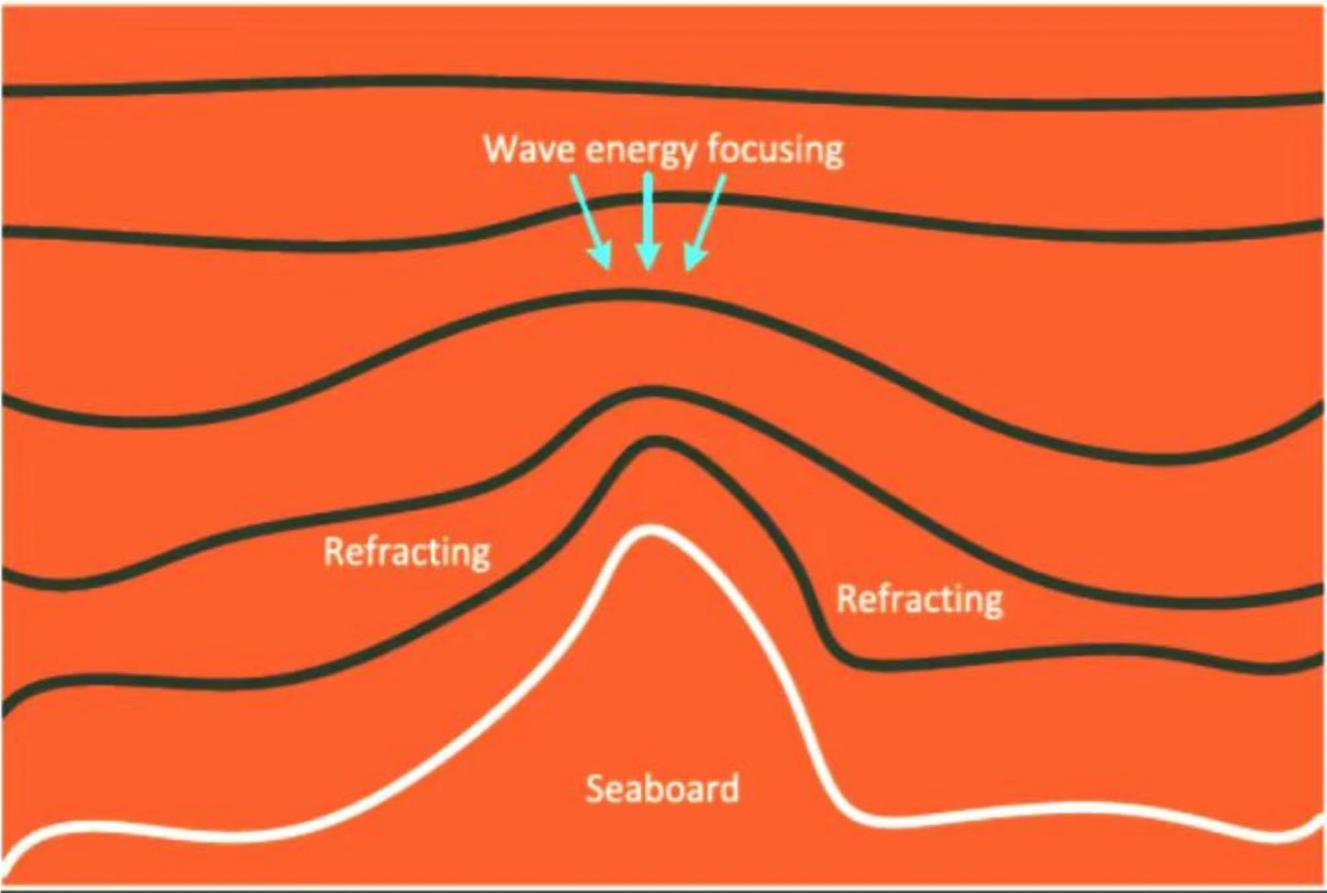
Wave refraction phenomena^44^.

Where, after the propagation, which occurs during each iteration, each value should propagate to obtain a new optimal value and shown by $\:{x}^{{\prime\:}}$. *L(t)* is the length of $\:{t}^{th}$dimension of search space (1 ≤ *t* ≤ n) and uniformly distributed random number within the range of [-1, 1]. r is the reduction coefficient and is very small positive number to avoid to get the infinity output. $\:{f}_{max}$ and $\:{f}_{min}$ are the maximum and minimum fitness numbers at presesent while $\:\epsilon\:$ denotes the least positive number to provision the divion by zero. Once the propagation is done next step is to find the refraction of cost and it includes the Gaussian random number with mean and standard deviation in (21–23) and refraction is illustrated in Fig. 3.

21 $${x}^{{\prime}} (t) = N (\mu, \sigma)$$

22 $$\mu = \left(\frac{{x}^{*}\left(t\right)\:+\:x\left(t\right)}{2}\right)$$

23 $$\sigma = \left(\frac{|{x}^{*}\left(t\right)-\:x\left(t\right)|}{2}\right)$$

Where *N (µ*,* σ)* denotes the gaussian random number, µ is the mean value and σ denotes the standard deviation. Meanwhile, after the refraction, the closeness of result may be reset by the wavelength.

24 $$\:{\lambda\:}^{{\prime\:}}\:=\:\lambda\:\:\frac{f\left(x\right)}{f\left({x}^{{\prime\:}}\right)}$$

**Fig. 4:**
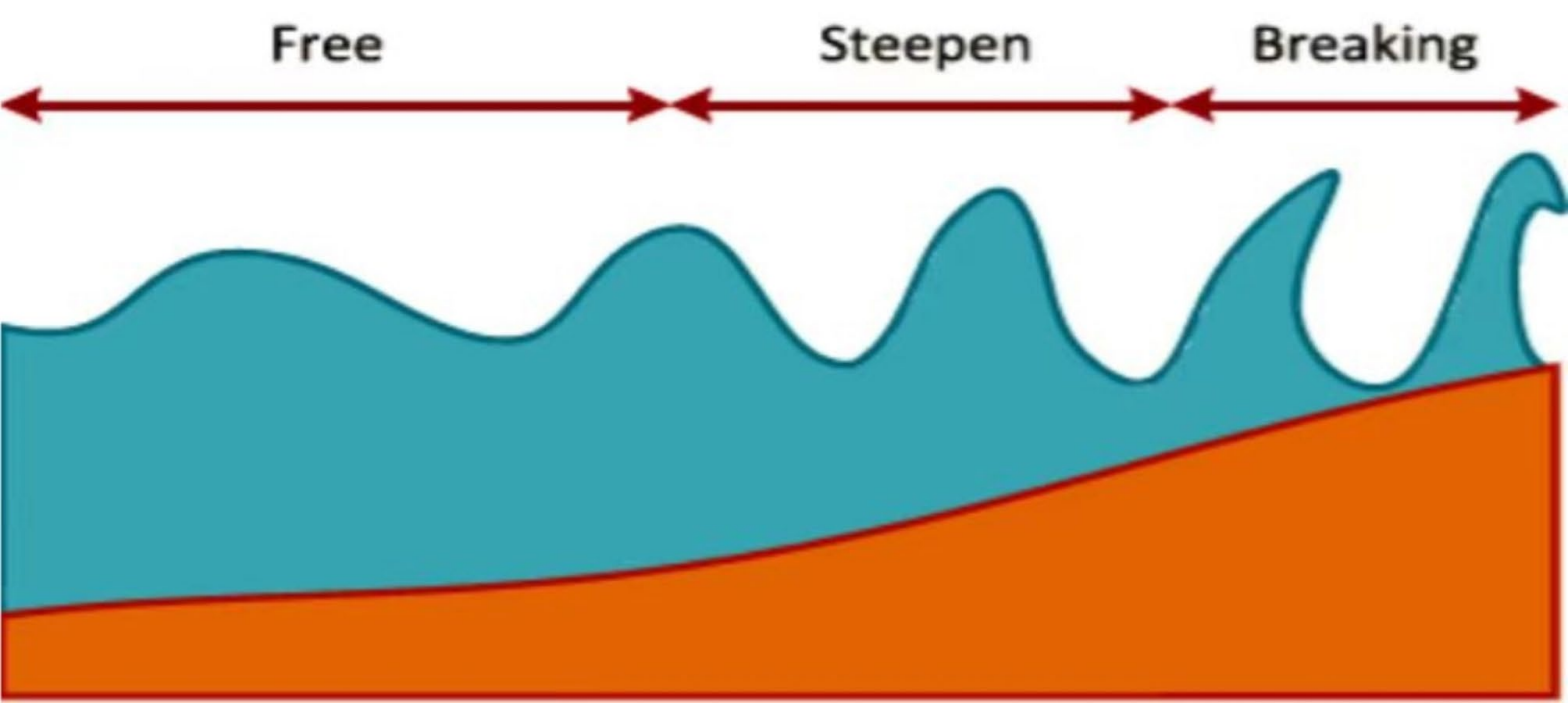
Breaking phenomena^44^.

Finally, Breaking operator regulates a centralized search around the optimal results and eventually solutions in the wave transforming into a series of individual waves elaborated in (24) and it can depicted from Fig. 4.

25 $${x}^{{\prime}}(t)=x(t)+N(0,1) \cdot \beta L(T)$$

β is the breaking coefficient and which ranges between 0.001 and 0.01.

A simple linear model has been suggested that decreases the inhabitants of n waves from maximum size $\:{iteration}_{max}$ to smallest value $\:iteration$ as described in (26). The adaptive wavelength coeffeient helps to increase the efficiency of propagation can be measured in (27–28).

26 $$n = \text{iteration}_{\rm max} - (\text{iteration}_{\rm max} - \text{iteration} )\frac{\:{\text{n}}_{\text{C}\text{G}\text{N}}}{\:{\text{n}}_{\text{M}\text{G}\text{N}}}$$

27 $$\text{r}(\text{iteration})=\text{r}_{\rm max}\cdot (\frac{{\text{i}\text{t}\text{e}\text{r}\text{a}\text{t}\text{i}\text{o}\text{n}}_{\text{m}\text{a}\text{x}}-\text{i}\text{t}\text{e}\text{r}\text{a}\text{t}\text{i}\text{o}\text{n}+1\:}{{\text{i}\text{t}\text{e}\text{r}\text{a}\text{t}\text{i}\text{o}\text{n}}_{\text{m}\text{a}\text{x}}}{)}^{{\uptheta\:}}$$

28 $$\theta = \frac{\text{log} \ ({\text{r}}_{\rm min}/{\text{r}}_{\rm min})}{\text{log}(1/\text{iteration}_{\rm max})}$$

Where, $\:{r}_{min}$ and $\:{r}_{max}$ are the minimum and maximum reduction coefficients while the existing production is denoted by the iteration of process. In the preliminary steps, r enhances exploration by having higher values. As the number of iterations rises, r decreases, leading to improve exploitation performance. Therefore, it strikes a favorable balance between exploration and exploitation.

Whereas the constraints of the optimization problem encompass factors like the power output from each type of Distributed Energy Resource (DER), preserving power balance within the Microgrid (MG) system, and managing energy exchange between components. The constraints on power generation for each type of DER include both the minimum and maximum output power confines at any given time t and can measured as in (29).

29 $$P_{minimum} \ {\leq} \ {P}_{output} \ {\leq} P_{maximum}$$

Where $\:{P}_{minimum}$and $\:{P}_{maximum}$ are the limits of output power is measured in watts, and $\:{P}_{output}\:$is the acceptable output power.

Table 1 provides an illustration of the significant differentiators between the PSO-MWWWO approach, and established microgrid design methods. These include comparisons with the widely used HOMER software, the conventional industry approach for powering critical loads, and numerous remarkable methods from cited sources^14,15^and^30,32^. The PSO-MWWO method drawn in this research incorporates all the essential elements that designers require when devising resilient and sustainable microgrid systems customized for applications.

table 1.

**Table 1:** Comparison of hydrogen based microgrid with traditional designs.

Design criterion	PSO-MWWO	HOMER	[14], [15]	[32], [34]
Components sizing	✔	✔	✔	✔
Renewable production	✔	✔	✔	✔
Hydrogen storage	✔			
Economy	✔	✔	✔	✔
Efficiency	✔		✔	✔
Reliability	✔		✔	
Voltage convergence	✔			

**Fig. 5:**
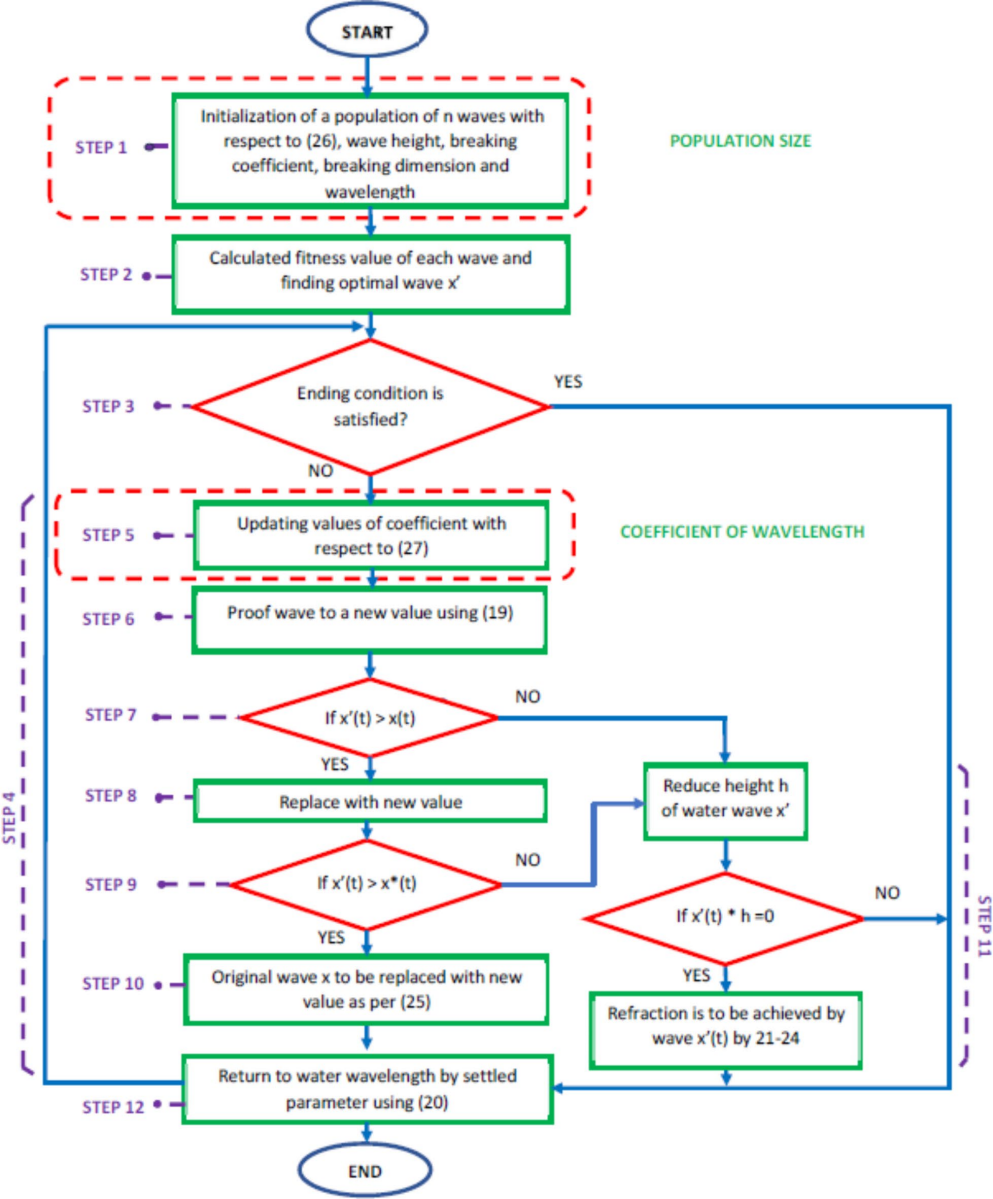
Flow chart for Bi-level optimized model for EMS of hydrogen based MG.

The proposed Energy Management Systems (EMS) contains the deliberation of multiple aims, and comprises technical and economic features. As far as the technical point, EMS aims to optimize equipment life and reduce power losses during the supply to loads by improving the system efficiency. whereas, the cost-effectiveness of a system is a primary concern once the economics analyses. Various constraints are integrated into this EMS architecture, including the inclusion of hydrogen energy storage, handling excess power during hydrogen generation, conducting power demand from utility, and adhering to time constraints. The proposed flow chart outlining the MWWO is described in Fig. 5.

## Simulation and results

In this study, the aforementioned stages need to be executed by engaging the EMS and a power that keeps regular surveillance of the system’s status and governs power distribution. To address the optimization trial, the Yalmip toolbox is utilized^46^. Meanwhile, the article adopts the bi-level optimization that is Modified Water-wave optimization MWWO and Particle Swarm Optimization (PSO) technique, commonly known by PSO-MWWO. It is implemented for its ability to efficiently and accurately tackle complex problems within a short timeframe. This optimization approach has been integrated into a MATLAB Simulink model.

In this research, the simulation development takes several inputs into account, including economic data, daily solar radiation, wind speed measured in meters per second, and the electricity demand from consumers^47^. The assumptive peak load for consumers within the microgrid is about in MW. Table II offers the average solar radiation, wind speed (in per unit), and the load characteristics. The capital costs for each unit are attained from^48^ and summarized. The exploration reinforces a forecasted technique that blocs the modified water wave optimization (MMWO) and Particle Swarm Optimization (PSO) with microgrid system applications within the MATLAB software environment. The optimal sizes for each unit in the sense of cost are detailed in Table II.

**Table 2:** Characteristics of multiple resources per day.

Wind speed (m/sec)	Solar radiation (W/$\:{\varvec{\text{m}}}^{2}$)	Electrolyzer kW	$\:{\mathbf{\text{H}}}_{2}$Igen	Load (p.u)
6.4	422	5	300/1/25	0.6211
Capital cost of system				
Wind system ($/kW)	Solar panel ($/kW)	Electrolyzer ($/kW)	Fuel cell’s ($/kW)	
950	1000	150	600	
Optimized cost of system per day				
Wind system ($/kW)	Solar panel ($/kW)	Electrolyzer ($/kW)	Fuel cell’s ($/kW)	
670	700	440	900	

**Fig. 6:**
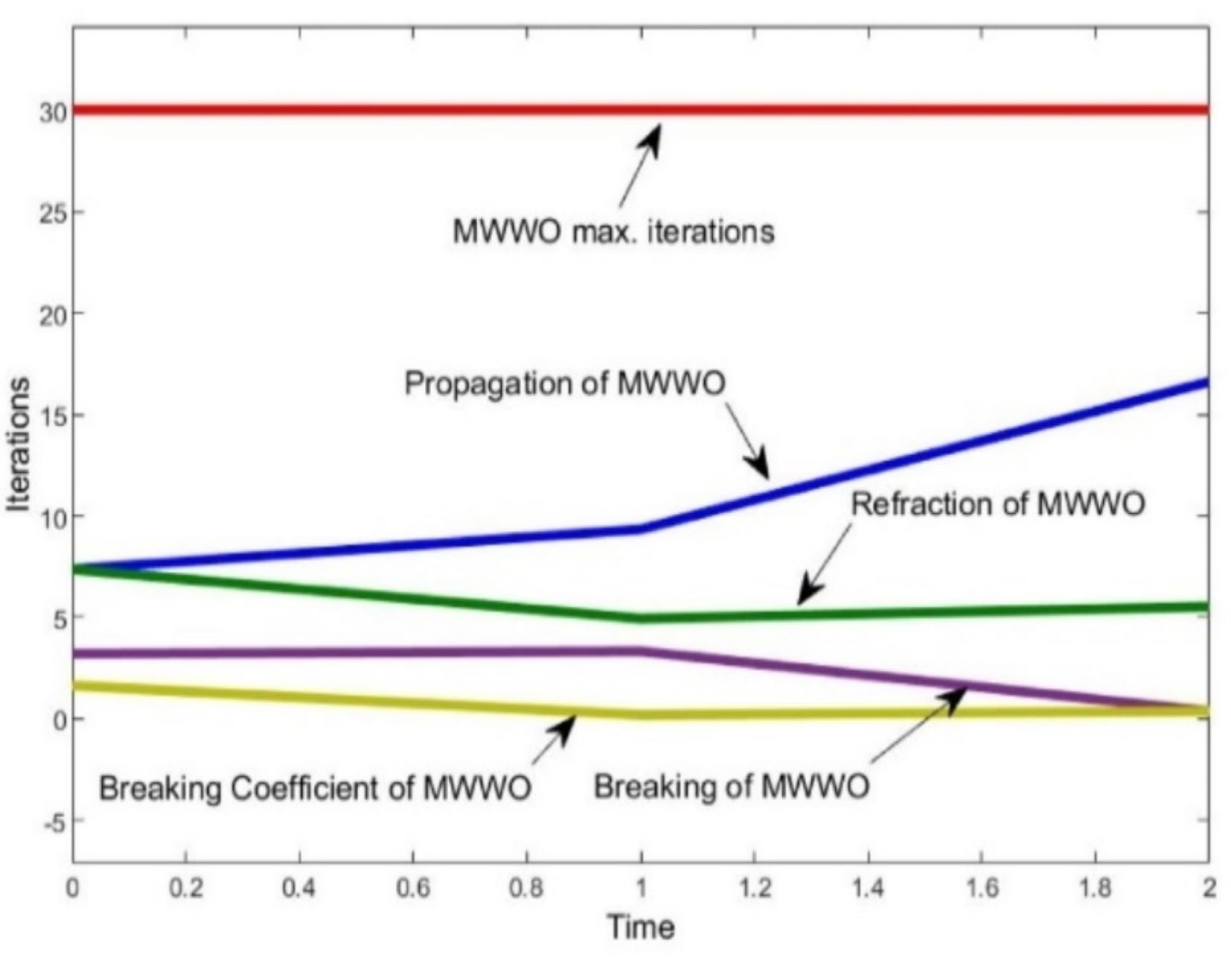
Simulated curves for PSO-MWWO

**Fig. 7:**
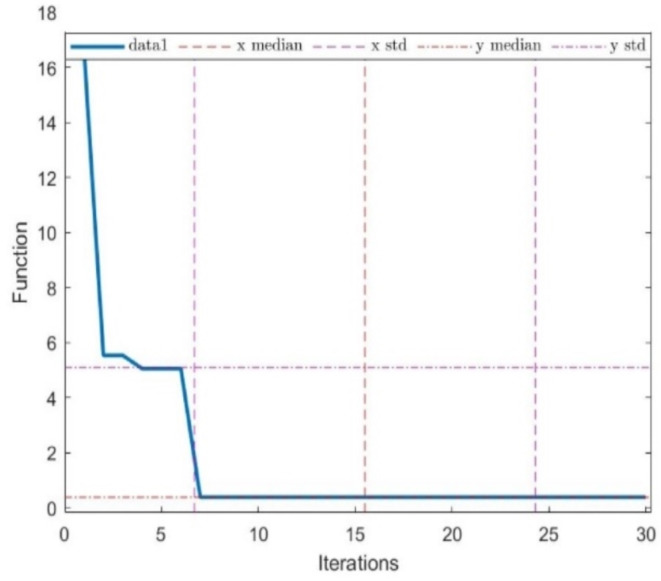
Optimized Fuel cell characteristics.

The operational categories of MWWO as revealed in Fig. 6. The convergence etiquette of the MWWO algorithm regarding cost, which leads to the optimal strategy for the hydrogen based microgrid system, is interpreted as in Fig. 7. The cost dynamically decreased for the hydrogen based microgrid systems.

The optimal solution is achieved remarkably after just thirty iterations. The power generated from the solar system, wind turbine, and fuel cells is effectively utilized to meet the consumers’ demands. The second phase of the research comprises the voltage convergence of hydrogen based microgrids. It’s a bilevel optimization implementation so here PSO is engaged. Once the power production is done from different renewable energy resources then a crucial part is occupying the fuel cell through electrolyzers. They are extensively used for the separation of water into its constituent elements, hydrogen and oxygen. Electrolyzers have various applications, including hydrogen production for fuel cells, energy storage, and contributing to the development of a more sustainable and renewable energy ecosystem. In a fuel cell, hydrogen combines with oxygen to produce electricity and water as a byproduct, making it a clean and efficient method of generating electric power. The voltage level of each renewable energy resource plays a vital role in meeting load with reduced uncertainties, as the voltage and power profile are also considered in this part of the article.

For validation, a vigorous voltage profile at the core generation source, the complete storage system can reap benefits such as amended battery state of charge and fuel cell current values. The fuel cell preserves a sustainable current to meet load during periods of RES power shortages, as depicted in Fig. 7. Furthermore, addresses fuel consumption during fuel cell energy management, which is presented in its corresponding characteristics. Fuel utilization within the energy management of fuel cells is another significant facet, which is comprised in its characteristics.

**Fig. 8:**
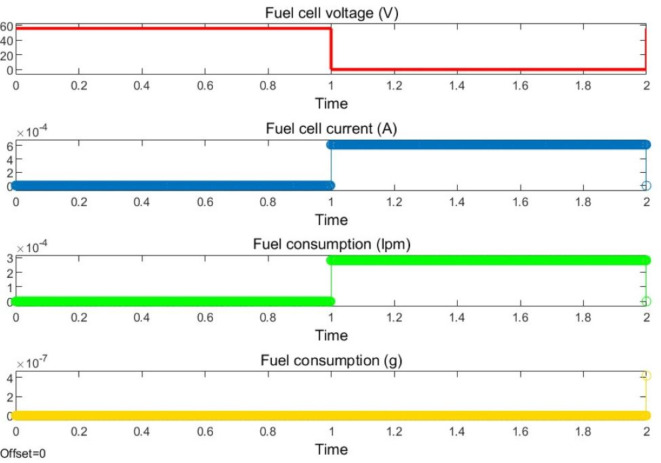
Optimized Voltages of hydrogen base microgrid.

**Fig. 9:**
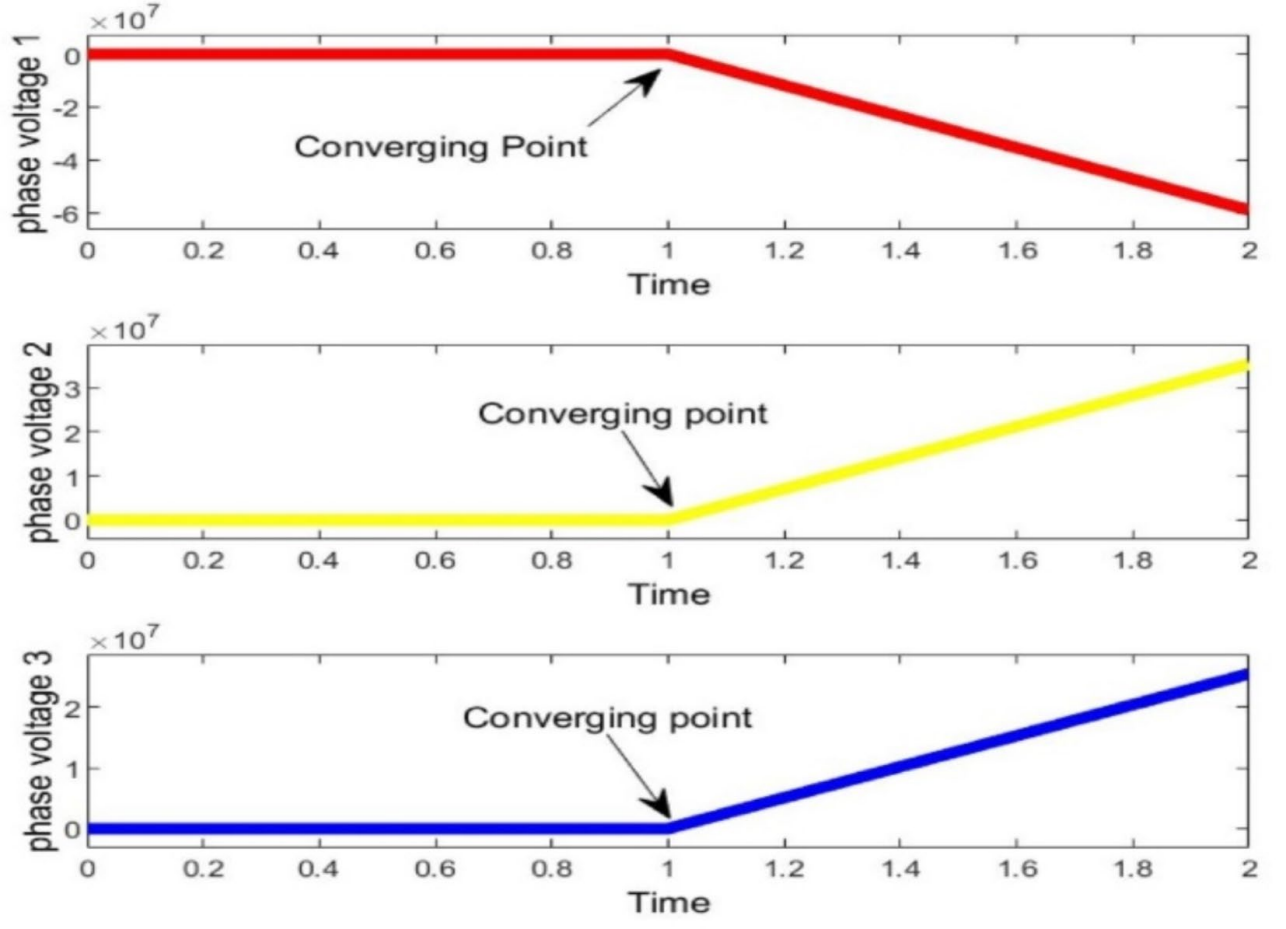
Voltage convergences of all phases of hydrogen based microgrid.

**Fig. 10:**
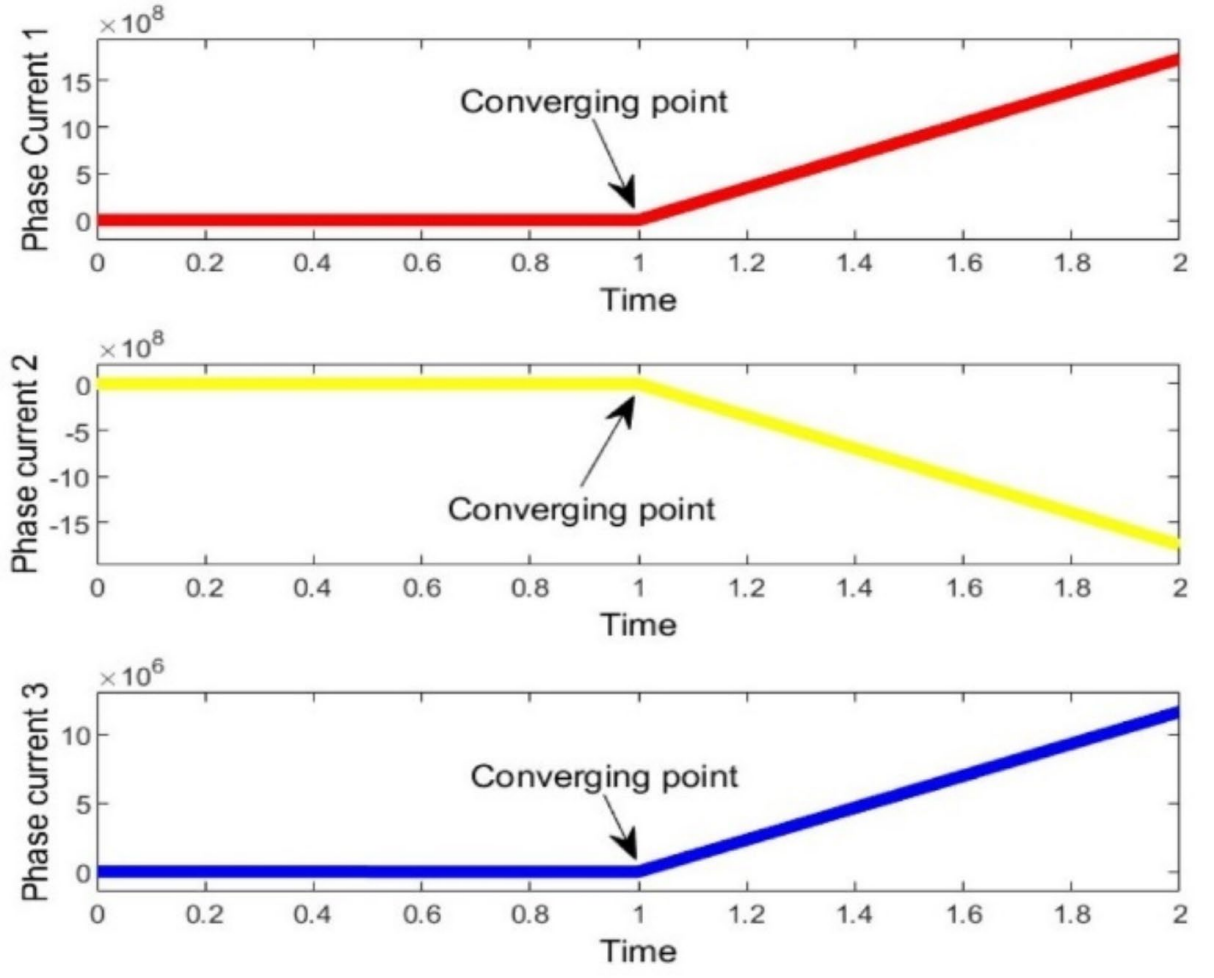
Optimized output of system.

It is evident that, in terms of optimization speed, both the MWWO and PSO algorithms closely approach the optimal solution after just 10 iterations, while the particle swarm algorithm requires at least 30 repetitions to attain a similar level of proximity to the optimal solution when its applied distinctly. When considering optimization outcomes, the model of bi-level algorithm emerges as the most favorable, presenting a more desirable optimal value for the capacity model compared to PSO. Optimized behavior of output voltage and current of multiple phases from simulation are in Figs. 8–10.

Finally, to assess the variability of the microgrid design generated by the PSO-MWWO method under diverse load conditions, a sensitivity analysis is conducted by examining the outcomes achieved in various voltage convergences. Figure 9 observes all voltage convergences, indicating that convergence was attained within the designed thirty iterations. Moreover, the converged voltage adhered to an encoded constraint, ensuring that it remains within the range of -2 to 2 with respect to their phases. The convergence part of the hydrogen based microgrid is implemented by means of PSO, resulting in an improvement of the power profile and system stability. The optimized output of the system is shown in Fig. 10. after the convergence of the entire system.

Predicting the voltage profile of a hydrogen based microgrid is a complex task due to its dependence on several factors, including microgrid accessibility. Moreover, critical loads often suffer changes in their installed capacity, resulting in disparities between the estimated power demand and power production.

**Fig. 11:**
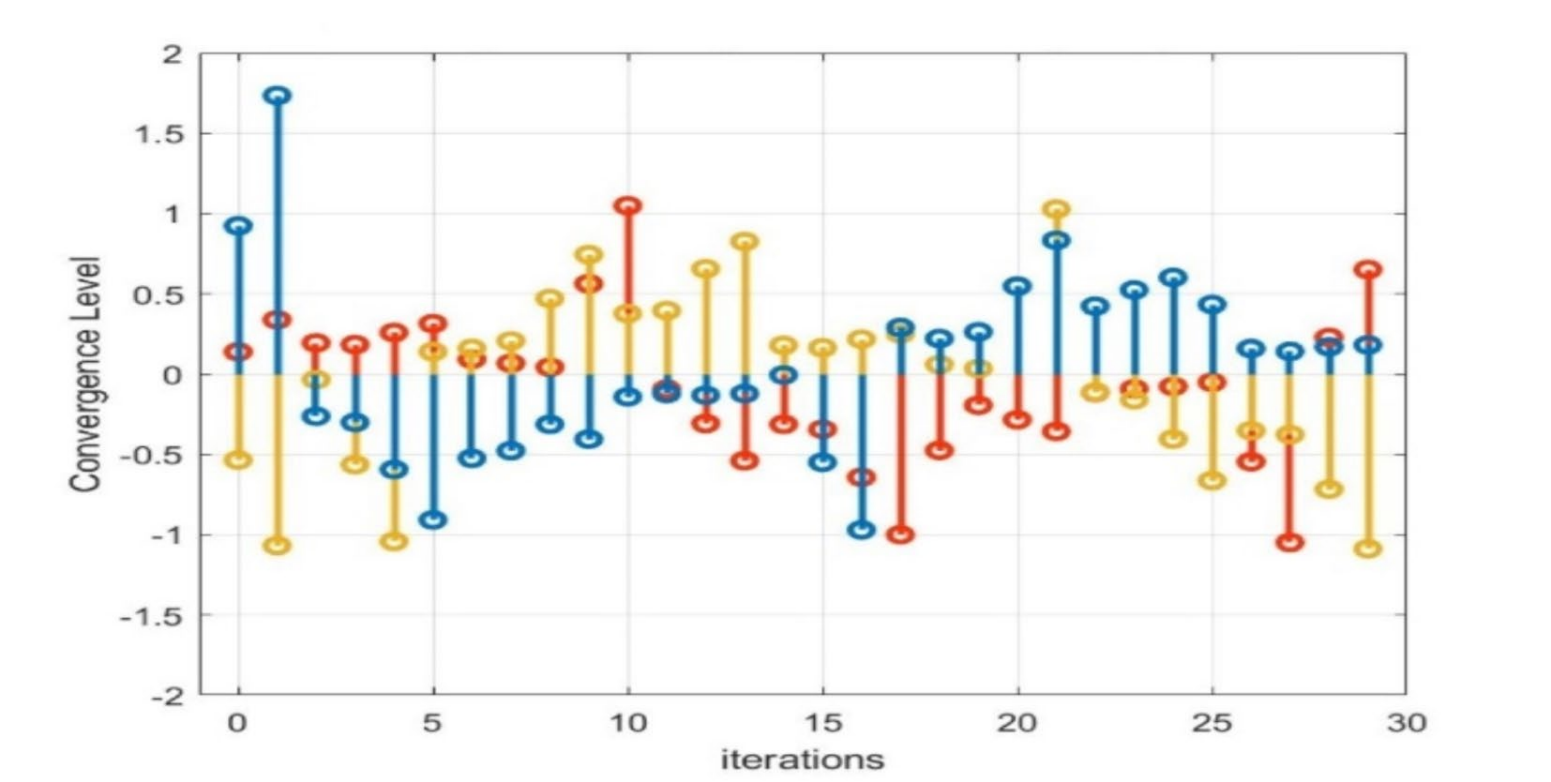
Sensitivity analysis for the cost of system.

**Fig. 12:**
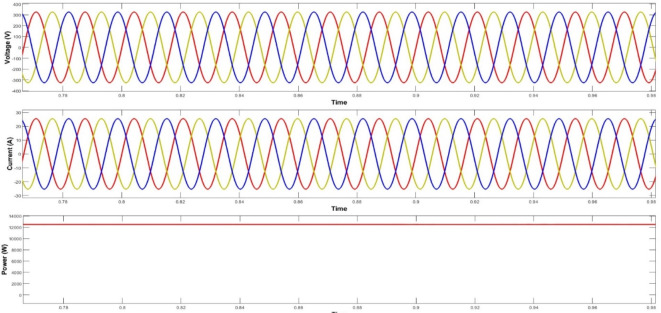
Sensitivity analysis of power quality.

The sensitivity analysis of the system is evaluated based on two key factors: (i) ensuring cost-effectiveness and (ii) improving power quality through the proposed technique. The cost sensitivity analysis examines how changes in parameters such as hydrogen storage efficiency, electrical load, and renewable energy integration impact the total system cost, as shown in the Fig. 11. The analysis reveals that while the costs for solar and wind energy are relatively similar, hydrogen storage and electrical load costs differ significantly from the costs of renewable energy resources. The observed pattern in the system cost will differ when there are variations in the electric load profile. Specifically, the system cost value increases as the electric load increases. The minimum and maximum total cost assessment can be derived from this analysis, revealing a range of approximately $2.17 million to $2.29 million. Figure 12. illustrates the sensitivity analysis concerning power quality improvement. Based on the calculations, wind velocity changes by approximately 49%, solar radiation varies by about 17%, and streamflow shows a variation of around 32%. This analysis examines the voltage deviations occurring over a 24-hour period, highlighting their significant impact on power quality.

Finally, the economic and policy factors interrelated with the hydrogen based microgrid must be calculated. Moreover, comparison of proposed PSO-MWWO with other traditional techniques on daily basis are shown in Fig. 14. It covers assessing the costs and benefits of the microgrid system and developing a technical model that can certify the economic viability of the system. The analysis underscores the effectiveness and viability of the PSO-MWWO in addressing the optimized capacity model for the hybrid hydrogen based microgrid proposed in this study. Even within this framework, the particular best hydrogen based microgrid design upholds an availability level that surpasses the established constraint. However, when confronted with heightened load impulsiveness, both the microgrid’s net present cost and energy efficacy experience a decay. These performance setbacks arise from the fluctuations in hourly power demand, causing a shift in the operating point of power converters towards a less efficient range.

PSO-MWWO exhibits exceptional global search capabilities by simulating natural evolutionary processes. It has proven effective in addressing a range of complex optimization challenges, encompassing both single-objective and multi-objective optimization problems. Traditional algorithms frequently struggle to achieve global optimal solutions. While metaheuristic algorithms do not guarantee a global optimum, they have a higher likelihood of finding it due to their mechanisms, such as crossover and mutation. PSO-MWWO does not depend on the gradient of the optimization problem, meaning it can handle problems that are not differentiable. This contrasts with traditional optimization methods like gradient descent and quasi-Newton techniques, which require differentiability. Time complexity is a fundamental aspect of the proposed research, as it gauges the amount of time an algorithm needs to complete based on the size of the input. It provides a structured framework for evaluating and comparing the efficiency of different algorithms. In this study, it was found that less time was required for both cost estimation and improving the power quality of the hybrid microgrid.

**Fig. 13:**
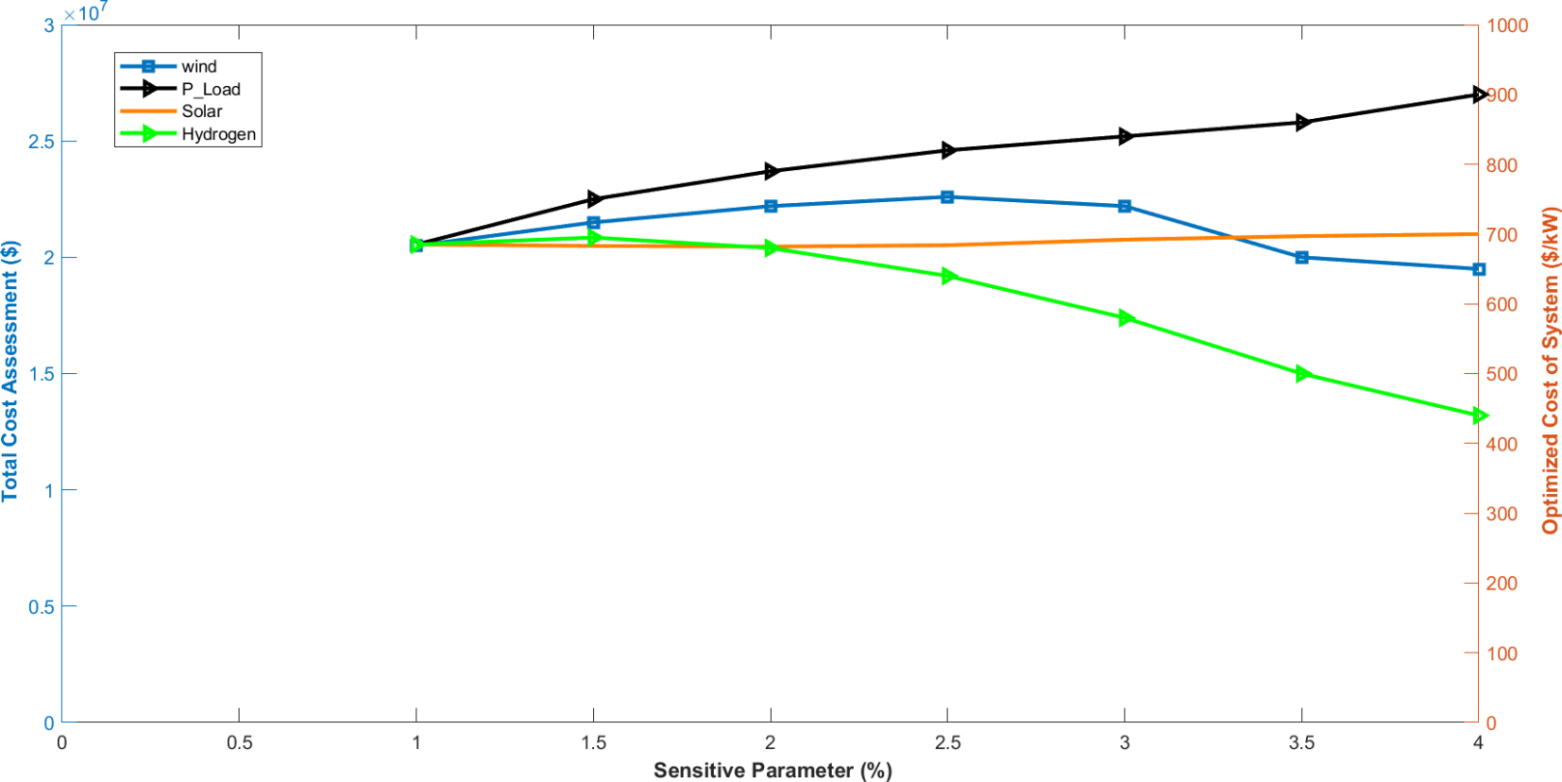
Sensitivity analysis of power quality.

**Fig. 14:**
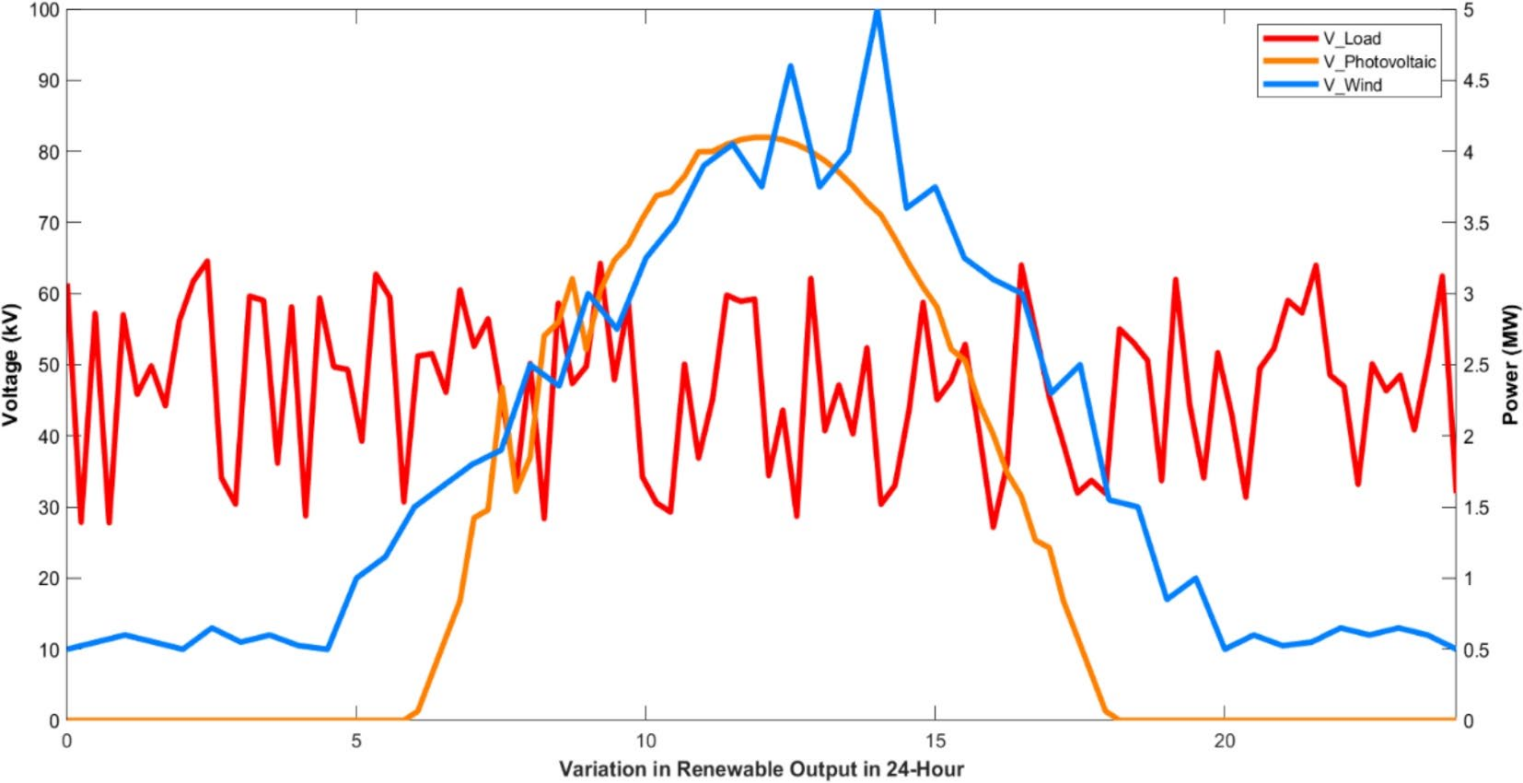
Sensitivity analysis of power quality.

**Fig. 15:**
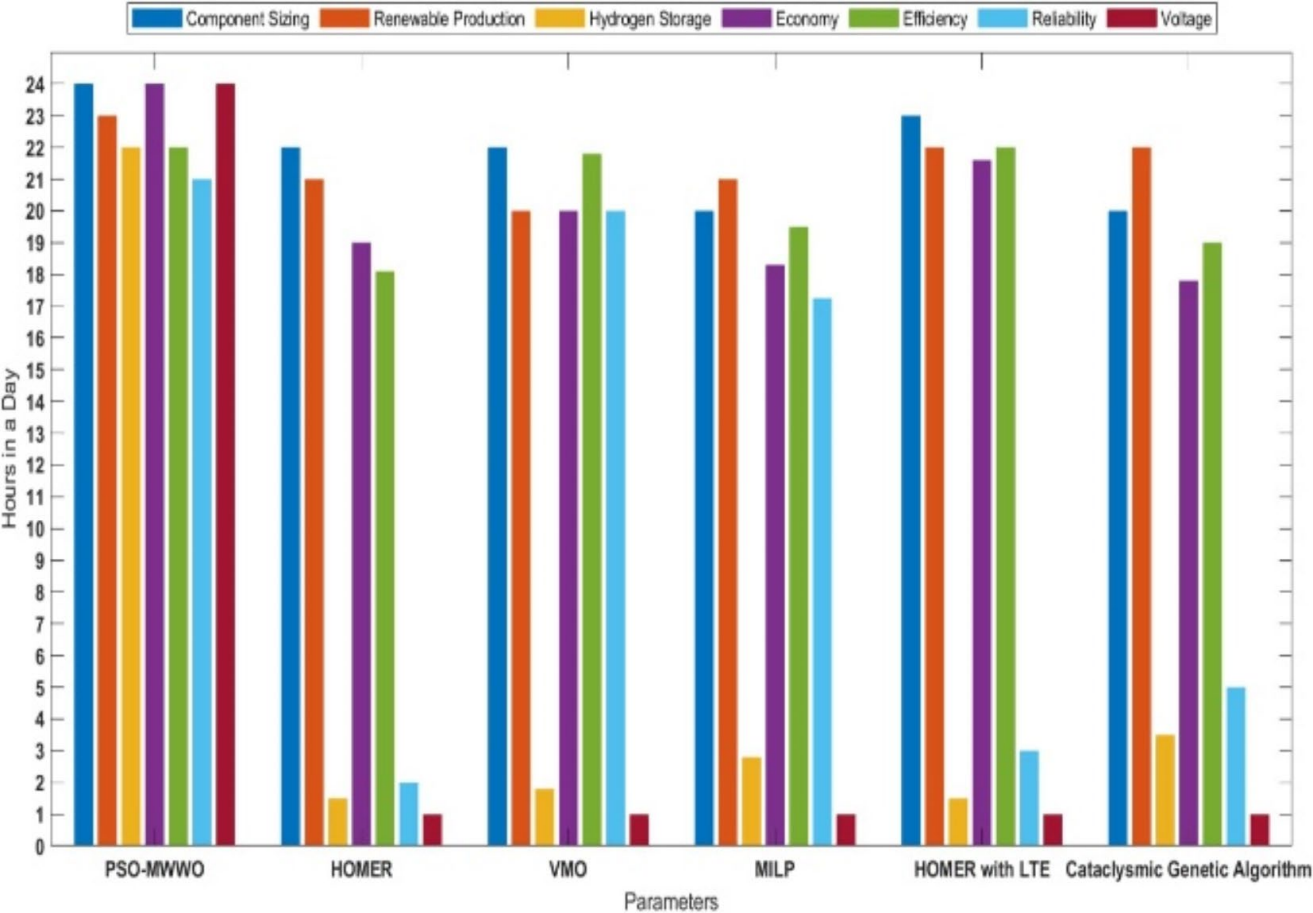
Comparison of PSO-MWWO with traditional techniques on daily basis.

The comparison of this proposed method with classical and contemporary methods has been conducted on a daily basis across multiple parameters in Fig. 15. This highlights the superiority of the proposed method over traditional methods already adopted in the field of hybrid microgrids. The considered parameters for comparison include component sizing, power production from renewable resources, hydrogen storage through electrolysis, cost-effectiveness, system reliability, and voltage convergence levels aimed at improving the power quality of the system. The proposed bi-level optimization technique PSO-MWWO has demonstrated highly commendable results, surpassing traditional methods with outstanding improvements in power quality and cost-effectiveness.

## Conclusion

This article introduces a novel approach for hydrogen based microgrids that serve for cost-effectiveness and improvement in power quality, which is ignored by the other scholars. The proposed approach tackles the shortcomings of conventional hydrogen based microgrid designs by integrating the selection of energy conversion structures and the sizing of energy sources into an integrated design framework. The planning process incorporates performance metrics like cost and power quality. Compared with other conventional researches on daily basis and it is found that its much advantageous. This article also provides a strategy for power convergence to validate the efficacy of this new technique. Through simulation, the assessment shows that power convergence enhances the quality of hydrogen-based microgrid systems, resulting in improved performance for hybrid microgrid regarding cost-effectiveness. The proposed method achieved savings of approximately 6.867% of the total cost by incorporating bi-level optimization. With a focus on power quality, this study has realized an impressive 94.9% improvement in cost-effectiveness by using bi-level optimization compared to previous methodologies.

Future research will concentrate on optimizing power delivery to consumers in hydrogen based microgrids, aiming to enhance society’s access to carbon-free energy sources. This paper has established the significance and engineering utility of System-based energy management for hydrogen based microgrids. Moreover, it lays the foundation for prospective research in the future. For instance, it opens the door for comprehensive optimization of both the microgrid system’s sizing and operation, which should be derived from the energy management methodology introduced in this study.

## Electronic supplementary material

Below is the link to the electronic supplementary material.

Supplementary Material 1

## Data Availability

The datasets used and/or analysed during the current study available from the corresponding author on reasonable request.

## Declarations

### Use of AI tools declaration

The authors declare they have not used Artificial Intelligence (AI) tools in the creation of this article.
