# Author Correction: Assessment of energy management and power quality improvement of hydrogen based microgrid system through novel PSO-MWWO technique

**DOI:** 10.1038/s41598-025-88770-2

**Published:** 2025-02-06

**Authors:** Hafiz Ghulam Murtza Qamar, Xiaoqiang Guo, Ehab Seif Ghith, Mehdi Tlija, Abubakar Siddique

**Affiliations:** 1 Key Laboratory of Power Electronics for Energy Conservation and Motor Drive of Hebei Province, Department of Electrical Engineering, Yanshan University, Qinhuangdao, 066004 China; 2 Department of Mechatronics, Faculty of Engineering, Ain shams University, Cairo, 11566 Egypt; 3 Department of Industrial Engineering, College of Engineering, King Saud University, P.O. Box 800, Riyadh, 11421 Saudi Arabia; 4 Department of Electrical & Biomedical Engineering, Khwaja Fareed University of Engineering and Information Technology (KFUEIT), Rahim Yar Khan, 64200 Pakistan

Correction to: *Scientific Reports* 10.1038/s41598-024-78-153-4, 05 January 2025

In the original version of this Article Mehdi Tlija was incorrectly affiliated with ‘Department of Electrical & Biomedical Engineering, Khwaja Fareed University of Engineering and Information Technology (KFUEIT), Rahim Yar Khan 64200, Pakistan’. The correct affiliation is listed below.

Department of Industrial Engineering, College of Engineering, King Saud University, P.O. Box 800, Riyadh 11421, Saudi Arabia.

The original Article has been corrected.

