# Supplementary material for: Assessment of energy management and power quality improvement of hydrogen based microgrid system through novel PSO-MWWO technique
Source: Sci Rep. 2025 Jan 5;15:863. doi: 10.1038/s41598-024-78153-4 (PMC11701170; doi:10.1038/s41598-024-78153-4)
Supplement: Supplementary file 1 — Supplementary Material 1 [file 41598_2024_78153_MOESM1_ESM.docx]

**Appendix 1**

**Design parameters of the system**

| Parameter | Symbol | Value |
| --- | --- | --- |
| Wind turbine speed | *Ns* | 15 rad/s |
| cut-in velocity | $V_{C-in}$ | 2.5 – 3.5 m/sec |
| cut-off speed | $V_{C-off}$ | 20 – 25 m/sec |
| Solar Irradiance | *I* | 500 |
| Cell Temperature | $T_{Cell}$ | 45^0^ C |
| Rated output voltage of inverter | $V_{out\_inv}$ | 475 V |
| storage efficiency of tank | *k* | 60%-80% |
| System frequency | *f* | 50 Hz |
| System Switching frequency | $f_{Switching}$ | 40 kHz |
| capacitance of filter | *C* | 1.10 mF |
| Damping resistance of system | $R_{f}$ | 4.11 mΩ |
| Resonance frequency of system | $f_{Resonance}$ | 48.31 Hz |
| Settling time of PLL | $T_{PLL}$ | 1.75 msec |
| Wave length | *λ* | 0.55 |
| Search space | *t* | 1 to n |
| Least positive number | *ε* | 0.0010 |
| Breaking coefficient | *β* | 0.001 – 0.01 |
